# Local-Scale Drivers of Tree Survival in a Temperate Forest

**DOI:** 10.1371/journal.pone.0029469

**Published:** 2012-02-13

**Authors:** Xugao Wang, Liza S. Comita, Zhanqing Hao, Stuart J. Davies, Ji Ye, Fei Lin, Zuoqiang Yuan

**Affiliations:** 1 State Key Laboratory of Forest and Soil Ecology, Institute of Applied Ecology, Chinese Academy of Sciences, Shenyang, Liaoning Province, People's Republic of China; 2 Department of Evolution, Ecology, and Organismal Biology, The Ohio State University, Columbus, Ohio, United States of America; 3 Smithsonian Tropical Research Institute, Balboa Ancón, Republic of Panamá; 4 Center for Tropical Forest Science, Arnold Arboretum, Harvard University and Smithsonian Tropical Research Institute, Boston, Massachusetts, United States of America; USDA-ARS, United States of America

## Abstract

Tree survival plays a central role in forest ecosystems. Although many factors such as tree size, abiotic and biotic neighborhoods have been proposed as being important in explaining patterns of tree survival, their contributions are still subject to debate. We used generalized linear mixed models to examine the relative importance of tree size, local abiotic conditions and the density and identity of neighbors on tree survival in an old-growth temperate forest in northeastern China at three levels (community, guild and species). Tree size and both abiotic and biotic neighborhood variables influenced tree survival under current forest conditions, but their relative importance varied dramatically within and among the community, guild and species levels. Of the variables tested, tree size was typically the most important predictor of tree survival, followed by biotic and then abiotic variables. The effect of tree size on survival varied from strongly positive for small trees (1–20 cm dbh) and medium trees (20–40 cm dbh), to slightly negative for large trees (>40 cm dbh). Among the biotic factors, we found strong evidence for negative density and frequency dependence in this temperate forest, as indicated by negative effects of both total basal area of neighbors and the frequency of conspecific neighbors. Among the abiotic factors tested, soil nutrients tended to be more important in affecting tree survival than topographic variables. Abiotic factors generally influenced survival for species with relatively high abundance, for individuals in smaller size classes and for shade-tolerant species. Our study demonstrates that the relative importance of variables driving patterns of tree survival differs greatly among size classes, species guilds and abundance classes in temperate forest, which can further understanding of forest dynamics and offer important insights into forest management.

## Introduction

Forests are an important and substantial part of the terrestrial biosphere, providing a number of key ecosystem functions such as carbon sequestration, and water and nutrient cycling. Tree mortality plays a critical role in forests [1]. It can determine forest dynamics or succession, alter nutrient cycling, and further contribute to tree species coexistence [2]–[4]. Without a proper understanding of the patterns and determinants of tree mortality, our overall understanding of forest dynamics is severely hampered. However, studies of tree mortality are hindered by the fact that trees are long-lived and mortality rates are often low, particularly for mature trees, making it difficult to gather sufficient observations and to gain insights into the factors influencing mortality and survival [5]. Ecologists are often forced to make overly simplistic assumptions about tree mortality processes. For instance, tree mortality has been hypothesized to be random, with all trees having an equal probability of survival [6]. However, in various natural forest communities numerous studies have rejected this random mortality hypothesis [7]–[9]. This is because trees are sessile and tree mortality is likely to be strongly affected by intrinsic properties of individuals and by their local immediate neighborhoods.

Many studies have shown that size is an important intrinsic property of trees that strongly affects survival. The probability of survival is expected to increase as trees get larger and acquire reserves to withstand environmental stress [9]–[11]. In addition, larger trees have a competitive advantage over smaller trees due to the asymmetric nature of competition for light [12]. However, tree survival is known to vary by tree size and these relationships are known to vary by species [3], [13]–[19].

Local neighborhood conditions broadly include two major classes of factors, biotic and abiotic variables. The effects of biotic and abiotic factors represent two important explanations for species coexistence in ecological communities: frequency (or density) dependence and resource niche partitioning, respectively [20]–[21]. In tree communities, survival is commonly observed to be frequency- or density-dependent, based on several studies that modeled survival as a function of the number, size and identity of individuals in the local biotic neighborhood [5], [8]–[9], [22]–[28]. Specifically, trees tend to have a higher probability of mortality where conspecific neighbors are denser, closer, or proportionately more abundant than heterospecific neighbors [8], [23], [29]–[30]. Such negative effects of conspecific neighbors could result from strong intraspecific competition for resources or from host-specific natural enemies, such as herbivores and pathogens that are attracted by or spread rapidly through high density patches of susceptible individuals. Negative conspecific density and frequency dependence are widely recognized as prominent mechanisms of species coexistence, and several hypotheses, such as the Janzen-Connell hypothesis, consider their effects on community assembly [31]–[34]. Although there have been several studies of neighbor effects on tree survival, few have simultaneously considered the effects of both biotic and abiotic factors, although in some cases researchers have tried to select environmentally homogeneous areas [23], [35]. However, even local environments are heterogeneous due to variation in topography and soils [36]–[37]. Failing to consider the effects of these abiotic factors on plant performance can result in incorrect inferences about the importance of density-dependent neighborhood effects [8]. For example, a species will likely attain higher densities in its optimal habitat due to increased survival. As a result, survival could then be positively correlated with conspecific density, potentially masking negative impacts of conspecific neighbors.

Studies have also shown that species with different life history strategies respond differently to intrinsic and extrinsic factors. Shade tolerance can be a key trait in determining patterns of tree growth and survival [23], [38]. In addition to being less sensitive to shading by neighbors, shade-tolerant species are less susceptible to enemy attack than light-demanding species, based on differences in the allocation of resources to defense vs. growth [39]–[41]. As a result, shade tolerance should be an important determinant of trees' reactions to their local biotic neighborhood [42]. In addition, some studies have found that species abundance is related to species' responses to their neighbors. For example, rare tropical tree species have been shown to be more sensitive to the density of neighboring conspecifics [23], [43]. Within a species, tree size may influence the effects of biotic and abiotic variables on survival. Smaller individuals may be more sensitive to neighbor density, due to asymmetric competition with taller individuals. Smaller individuals may also be more likely to show effects of abiotic habitat variables on survival, since large trees are typically found in preferred habitats due to environmental filtering occurring at earlier life stages [44]–[45].

In this study we assessed the relative importance of tree size, abiotic conditions, and the local biotic neighborhood in driving patterns of tree survival in an old-growth temperate forest in northeastern China at three organizational levels (community, guild and individual species). We used data from multiple censuses of a large (25 ha), fully-mapped forest plot to address the following questions: (1) Does tree size have a consistent positive effect on tree survival? (2) Are abiotic or biotic neighborhood factors more important in influencing spatial patterns of tree survival? (3) Can life history strategy or ecological guild be used to predict the relative importance of factors driving tree survival?

## Materials and Methods

### Study area

The study area was the Changbai Nature Reserve, which extends along the border of China and North Korea from 127°42′ to 128°17′E and 41°43′ to 42°26′N. It is one of the largest biosphere reserves in China and has been spared from logging and other severe human disturbances. The Changbai Nature Reserve joined the World Biosphere Reserve Network under the UNESCO Man and the Biosphere Programme in 1980. The reserve is ca. 200,000 ha, and elevation ranges from 740 m to 2,691 m at the summit of Changbai Mountain.

Our study area is located in broad-leaved Korean pine (*Pinus koraiensis*) mixed forest, the most common vegetation type in the region. Mean annual precipitation is approximately 700 mm and most of this occurs from June to September (480–500 mm). Mean annual temperature is 2.8°C, with a January mean of −13.7°C, and a July mean of 19.6°C. Mean age of overstory trees is ca. 300 years. Common tree species include *P. koraiensis, Tilia amurensis, Quercus mongolica, Fraxinus mandshurica, Ulmus japonica*, and *Acer mono* [46].

### Field methods

A 25 ha (500×500 m) forest plot was established in the summer of 2004 in Changbaishan (CBS) Nature Reserve. The plot, representative of forests in the area, is located in the core zone of Changbai Nature Reserve. Mean elevation in the plot is 801.5 m and ranges from 791.8 m to 809.5 m. All free-standing trees at least 1 cm in diameter at breast height (dbh; 1.3 m above ground) were tagged, measured and identified to species, and their geographic coordinates were recorded following a standard field protocol [47]. The first CBS plot census started in July 2004 and ended in September 2004, and the second census was carried out between July 2009 and August 2009. The status of trees, as live or dead, was recorded in the second census. The CBS plot contains 52 species with stems ≥1 cm dbh, belonging to 32 genera and 18 families [46]. No specific permits were required for the described field studies.

### Biotic neighborhood variables

To quantify the local biotic neighborhood, we used the frequency of conspecific neighbors (basal area of conspecific stems/total basal area of all stems) and the total basal area of all stems ≥1 cm dbh within 20 m of the center of each focal tree. We chose 20 m because tree species interactions have been shown to disappear beyond 20 m [48].

### Abiotic factors

Elevation was measured at the corner of each 20 m×20 m quadrat in the 25 ha plot. Elevation values for these 20 m×20 m quadrats were interpolated to calculate the elevation of the corners of the 5 m×5 m subquadrats. Slope and aspect values were then calculated for each subquadrat.

In 2007, soils were sampled using a regular grid of points every 30 m. Two additional sample points at 2, 5, or 15 m were selected in a random compass direction from the grid point to capture variation in soil nutrients at finer scales. In total, 967 points were sampled in the 25 ha plot. At each sample point 500 g of topsoil (0–10 cm depth) was collected, and eight soil properties (pH, organic matter, total N, available N, total P, available P, total K and available K) were analyzed according to Lu [49]. Soil pH in water (1∶1) was measured by Beckman glass electrode. Soil organic matter was determined colorimetrically by the dichromate oxidation method. Total N was determined colorimetrically on the KCl extracts using the Kjeldahl method. Available N was alkali dispelled by 1 mol NaOH L^−1^. Total P was determined by molybdenum antimony blue colorimetry after extraction using H_2_SO_4_-HClO_4_. Available P content was extracted using a 0.05 mol/LHCL-0.025 mol/LH_2_SO_4_ solution. Total K was determined by digesting in hydrofluoric acid and then measured by atomic absorption spectrometer. Available K was extracted with 1 mol NH4AcL-1 and then measured by atomic absorption spectrometer.

Spatial predictions for 5 m×5 m quadrats were obtained using geostatistical methods (ordinary kriging). Because these soil variables were strongly correlated with each other, we computed the principal components (PCs) from the eight soil variables and used only the first two components as condensed variables because together they explained 86.6% of the total variance in soil variables (Table S1). Variable condensing can reduce the possibility of overfitting the models [37].

### Data analysis

We modeled the probability of an individual tree surviving the 5-year census interval as a function of initial tree size in the first census (i.e. dbh) and the abiotic and biotic neighborhood factors described above, using generalized liner mixed models (GLMM) with binomial errors with a logit link function (i.e. logistic regression). GLMM is now widely used in ecology [50] and details on GLMM can be found in Zurr *et al*. [51] Biotic factors included the total basal area of all neighboring trees and the frequency of conspecifics (basal area of conspecific stems/total basal area of all stems) within 20 m of the focal tree. For abiotic factors, we included three topographical variables (elevation, slope and aspect) and the two PC axes of soil variables. We assigned to each individual tree the abiotic factors of the 5×5 m quadrat where it was located. All abiotic and biotic variables, as well as log-transformed initial tree size (dbh), entered the model as fixed effects. For all these variables, values were standardized by subtracting the mean value of the variables (across all individuals in the analysis) and dividing by 1 standard deviation. This allows for a direct comparison of the relative importance of these explanatory variables [52]. The mean and range of all explanatory variables used in the analysis are listed in Table S2. To avoid edge effects, we excluded all potential target trees that were within 20 m of the edge of the plot from the analyses.

Tree survival was analyzed at three different scales. First, we included all trees in the plot and conducted a community-level analysis with all species. Species was included as a random effect in the analysis. Second, we assessed the effect of species life-history traits by dividing species into different life-history guilds and analyzing each guild separately, with species as a random effect. Previous studies in the CBS forest found that species distributions and interactions varied among different guilds [48], [53]. Therefore, we expected that the effects of explanatory variables on tree survival would also differ among guilds. Specifically, we classified species into three shade-tolerant guilds (shade-tolerant, mid-tolerant and light-demanding) [54]; we also grouped species into four abundance classes based on their abundance in the CBS 25-ha plot (very rare: 1–100, rare: 100–1000, common: 1000–5000, and very common: >5000 individuals). In addition, we divided individuals into three size classes (1–20 cm, 20–40 cm and >40 cm dbh). In these analyses, species was also included as a random effect. All explanatory variables were standardized as described above. Finally, because niche theory predicts that species will be affected differently by abiotic variables, we performed species-level analyses, by separately analyzing each of the 20 species with >100 stems.

To test the relative importance of tree size, abiotic variables and biotic variables in affecting tree survival, we constructed four candidate models with different variables: (1) only tree size; (2) tree size and abiotic variables; (3) tree size and biotic variables; (4) all variables included. We used Akaike information criterion (AIC) [55] to identify the best fit model, and then used the results from the best fit model to evaluate the effect and uncertainty of individual variables. Also, we validated the best fit models with Nagelkerke's 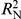 and Somer's 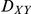 as measures of model predictive and discriminative ability, respectively. Nagelkerke's 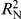 is a pseudo R^2^ (classical R^2^ is not appropriate for logistic models) to evaluate model predictions by calculating the square of correlation coefficient between observed and predicated values [56]–[57]. Somers' 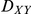 measures the correlation between predicted survival probability and a binary (0–1) variable [57]–[58]. The 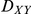 values range from −1 (where all live trees were classified as dead and vice versa) to 1 (all classifications were correct). All calculations were carried out in R version 2.10.0 [59], using the “lme4” package [60] with the Laplace method [50].

## Results

### Community-level analysis

**Figure 1:**
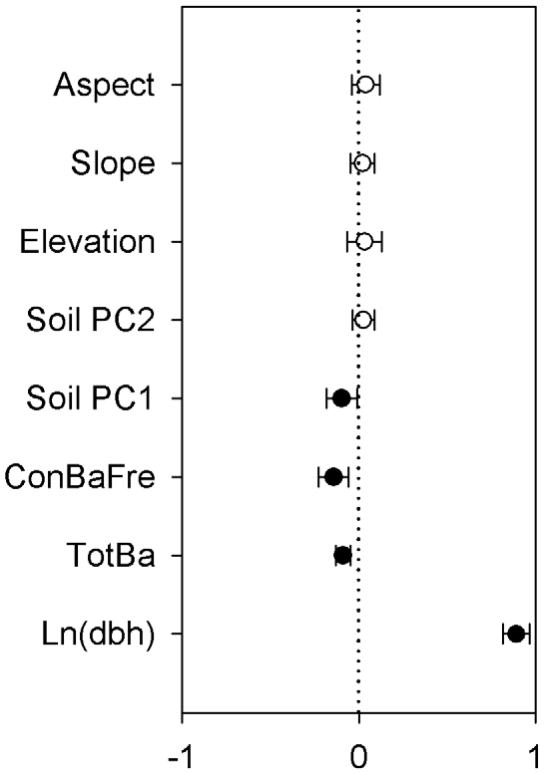
Standardized parameter estimates (±2 SE) of abiotic and biotic variables and size on tree survival for all species in the Changbai temperate forest. Filled circles indicate significant effects (*P*<0.05).

We assessed the role of tree size, the biotic neighborhood (i.e. frequency of neighboring conspecific basal area and total basal area of all neighbors), and local abiotic variables (i.e. soil properties and topography) on tree survival using generalized liner mixed models and compared the likelihood of models of increasing complexity to determine the best fit model. At the community-level, the model including all factors proved the best fit for tree survival (as indicated by the lowest AIC; Table 1), suggesting that tree size, abiotic and biotic factors all have significant effects on tree survival in the CBS temperate forest plot. However, low values of Nagelkerke's 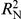 (0.118) and Somer's 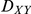 (0.452) (Table 2), measures of goodness of fit, suggest that much of the variation in probability of mortality remained unexplained. Of these influential factors, tree size (i.e. dbh) had the strongest impact on survival (Figure 1), with larger trees having an increased probability of survival. Frequency of conspecifics and total basal area of all neighboring trees within 20 m both had negative effects on survival. In addition, we found evidence that tree survival was influenced by soil factors (Figure 1). Specifically, the probability of survival was affected by soil PC1 (the first axis of a PCA using eight soil variables), which was associated with high concentrations of total K and low concentrations of organic matter and total N (Table S1). In contrast, topographic factors (elevation, slope and aspect) had no significant effects on tree survival (Figure 1).

**Table 1:** AIC values of the generalized linear mixed models of tree survival at the community and guild level.

Candidatemodels	All species	Abundance				Shade-tolerance			Tree size		
		Very rare	Rare	Common	Very common	Shade-tolerant	Mid-tolerant	Light-demanding	Small trees	medium trees	large trees
Size	19753.71	**580.55**	3519.88	6588.36	9053.79	16175.32	2053.64	1525.81	16798.93	**961.73**	577.96
Abiotic	19718.51	584.94	3519.56	6565.15	9029.77	16140.39	2058.27	**1523.01**	16769.66	**961.65**	586.13
Biotic	19712.22	**581.45**	**3512.13**	6583.34	9017.89	16148.04	**2050.55**	**1521.42**	16758.51	964.16	**570.11**
All factors	**19698.53**	585.89	3515.28	**6563.68**	**9008.16**	**16130.69**	2055.53	1523.50	**16745.5**	965.14	575.14

AIC values of the most likely models are shown in bold. Size, Abiotic, Biotic and All factors represent models with only tree size, tree size and abiotic factors, tree size and biotic factors, and all factors included, respectively. Very rare, rare, common, very common denote abundance classes with 1–100, 100–1000, 1000–5000 and >5000 individuals, respectively. Small, medium, and large tree size classes include individuals with 1–20 cm, 20–40 cm, and >40 cm dbh, respectively.

**Table 2:** Predictive and discriminative measures (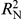 and 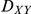) of the generalized linear mixed models of tree survival at the community and guild level.

	All species	Abundance				Shade-tolerance			Tree size		
		Very rare	Rare	Common	Very common	Shade-tolerant	Mid-tolerant	Light-demanding	Small trees	Medium trees	Large trees
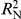	0.118	0.177	0.170	0.092	0.090	0.118	0.099	0.107	0.098	0.017	0.044
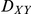	0.452	0.542	0.526	0.419	0.383	0.446	0.453	0.430	0.402	0.206	0.387

### Guild-level analysis

When analyzing different life history guilds separately, we found that the relative importance of factors influencing tree survival varied among guilds. Similar to the community-level analysis, the values of Nagelkerke's 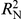 and Somer's 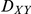 of the best fit model for each guild were relatively low (Table 2).

**Figure 2:**
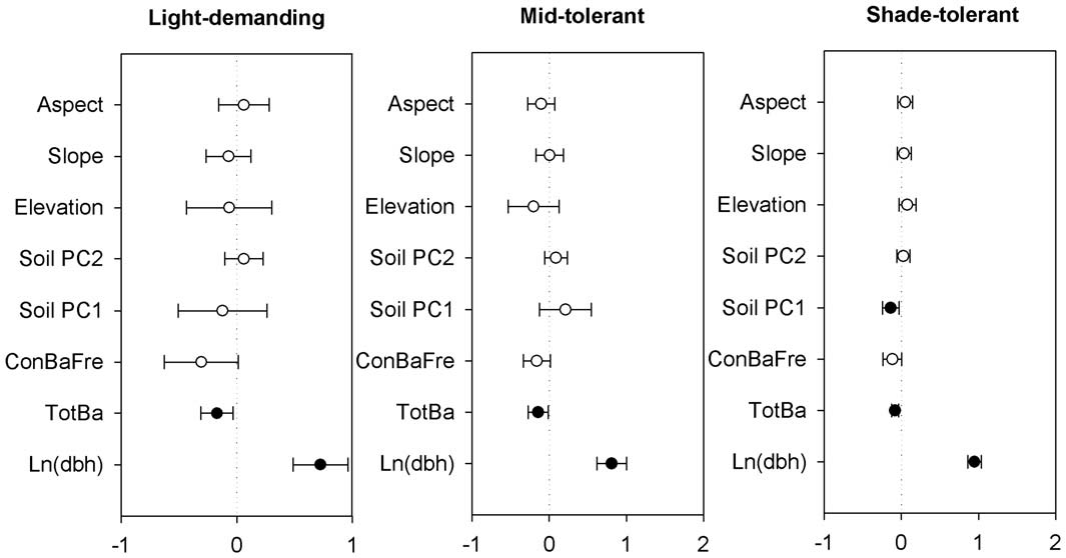
Standardized parameter estimates (±2 SE) of abiotic and biotic variables and size on tree survival for three shade-tolerant guilds in the Changbai temperate forest. Filled circles indicate significant effects (*P*<0.05).

Among the three shade-tolerance guilds (light-demanding, mid-tolerant and shade-tolerant), there were several differences in the relative importance of factors effecting tree survival. For the light-demanding group, the model with the lowest AIC only included tree size and biotic factors. That model could not be statistically differentiated from the model with tree size and abiotic factors (i.e. the difference in AIC was <2; Table 1), but none of the individual abiotic variables analyzed had a detectable effect on tree survival (Figure 2). The model with tree size and biotic factors was the best fit for the mid-tolerant group, while the model with tree size, biotic and abiotic factors was the best fit for the shade-tolerant group. Similar to the community-level results, tree size had the strongest positive effect on tree survival and total basal area had a significant negative effect for all shade-tolerance groups. Frequency of neighboring conspecifics had a marginally significant negative effect for all three groups (light demanding: *P* = 0.056; mid-tolerant: *P* = 0.063; shade-tolerant: *P* = 0.059). None of the topographic factors had a significant effect, while soil factors (Soil PC1) significantly affected survival for the shade-tolerant group (Figure 2).

**Figure 3:**
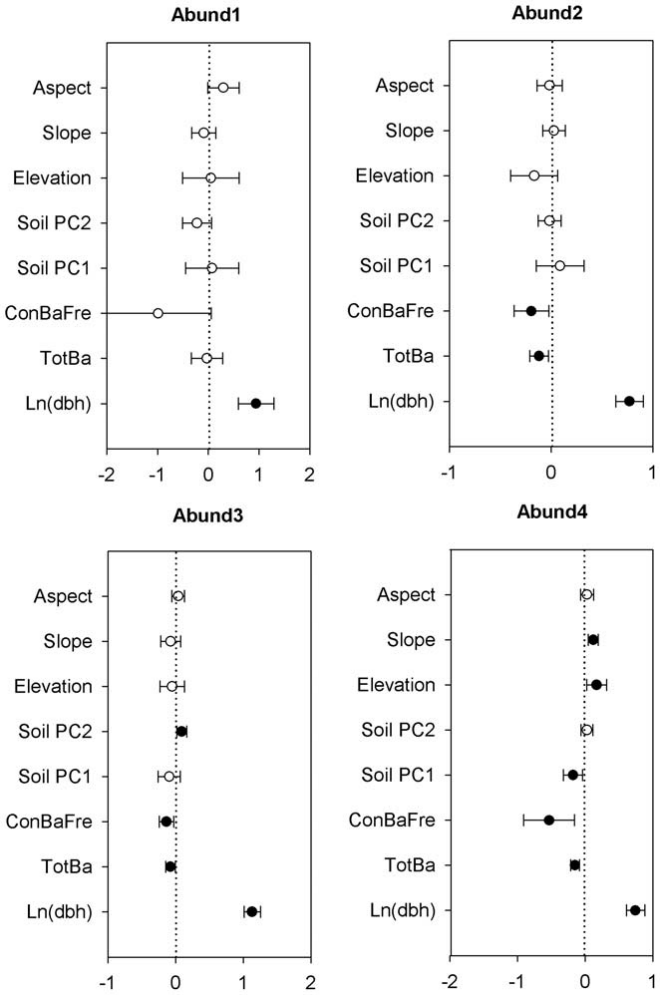
Standardized parameter estimates (±2 SE) of abiotic and biotic variables and size on tree survival for three abundance classes in the Changbai temperate forest. Filled circles indicate significant effects (*P*<0.05). Abund1, Abund2, Abund3 and Abund4 show abundance classes with <100, 100–1000, 1000–5000 and >5000 individuals, respectively.

We also fit models separately for four abundance classes (very rare, rare, common, and very common). For the abundance class with very rare species, two best fit models were found (Table 1). Both models included the effect of tree size, and one also included biotic factors. The model with tree size and biotic factors was the best fit for rare species. The best fit models for common and very common species included all factors. Among these best fit models, tree size had the strongest positive effect on tree survival. Frequency of neighboring conspecific basal area and total basal area had significant negative effects on survival of trees of rare, common and very common species (Figure 3). For the very rare class, the parameter value for conspecific neighbor frequency was more strongly negative than for the other classes, but was not significant. Effects of soil factors were found for common and very common classes, but topographical factors only contributed to survival for the very common class (Figure 3).

**Figure 4:**
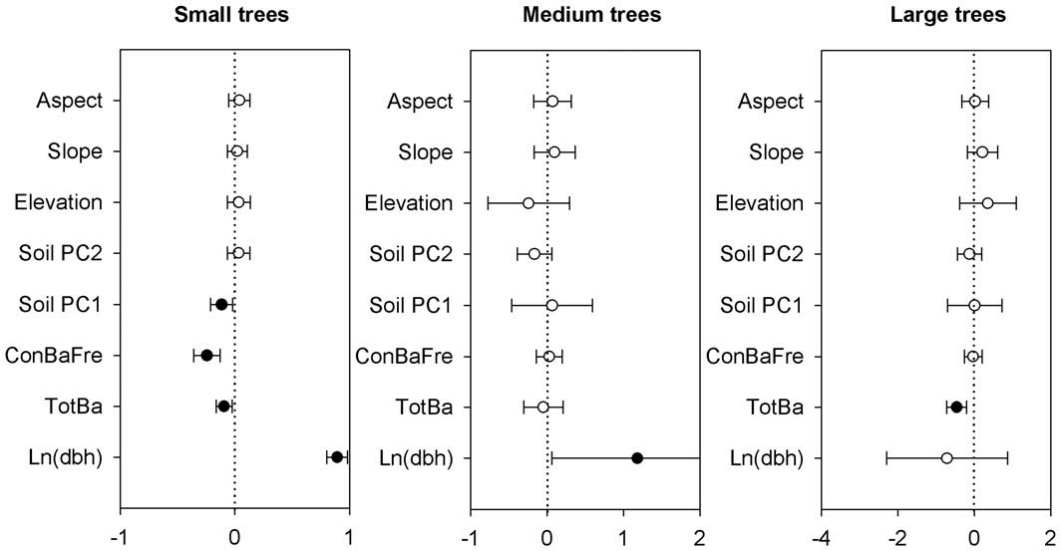
Standardized parameter estimates (±2 SE) of abiotic and biotic variables and size on tree survival for three tree size classes in the Changbai temperate forest. Small trees, Medium trees and Large trees show tree size classes with 1–20 cm, 20–40 cm and >40 cm dbh, respectively. Filled circles indicate significant effects (*P*<0.05).

The factors driving tree survival also varied when dividing individuals into three tree size classes (small, medium and large). For large individuals (dbh>40 cm), the best fit model included tree size and biotic factors, while the best model for the small size group (1–20 cm dbh) included all factors (Table 1). For medium trees (20–40 cm dbh), the best fit model included tree size, but it was not statistically different from the model that included tree size and abiotic factors (i.e. the difference in AIC was <2; Table 1). However, none of abiotic factors analyzed had a detectable effect on tree survival for the medium size group (Figure 4). In the three size groups, tree size did not always show strong positive effects on survival. For the small and medium size groups, survival increased strongly with increasing tree size. For the large size group, however, the relationship was slightly negative, but not significant. Total basal area of all neighboring trees had strong negative effects on tree survival for the small and large size groups, but frequency of neighboring conspecific basal area only had a significant negative effect on trees in the small size group (Figure 4). Topographic factors had no significant effects on tree survival for any of the three size groups, but the survival of small trees was effected by soil factors (Soil PC1; Figure 4).

### Species-level analysis

There were large differences in the best fit models for twenty species that were analyzed individually (Table 3). Values of Nagelkerke's 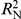 and Somer's 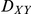 of the best fit models for these species differed greatly (Table 4). Nagelkerke's 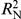 ranged from 0.026 (*Syringa reticulata*) to 0.304 (*Tilia mandshurica*). Somer's 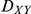 varied from 0.170 (*S. reticulata*) to 0.692 (*T. amurensis*), with 5 species higher than 0.6.

**Table 3:** AIC values of the generalized linear mixed models of tree survival for 20 tree species with >100 individuals.

Species	Size	Abiotic	Biotic	All factors
*Corylus mandshurica*	6271.48	6253.91	6247.28	**6240.24**
*Acer mono*	2739.86	2740.21	**2727.72**	2733.22
*Acer pseudo-sieboldianum*	1938.94	**1925.24**	1939.84	1929.05
*Acer barbinerve*	2017.28	**1994.27**	2010.67	**1993.12**
*Tilia amurensis*	793.10	**775.65**	790.39	779.42
*Pinus koraiensis*	**722.02**	725.47	724.87	728.51
*Syringa reticulata*	1070.24	**1066.53**	1069.44	1070.21
*Ulmus japonica*	519.25	519.63	**515.15**	**516.67**
*Quercus mongolica*	256.28	263.88	**253.26**	262.12
*Maackia amurensis*	**557.86**	561.53	**558.19**	562.47
*Fraxinus mandshurica*	175.68	180.16	**172.42**	176.23
*Acer tegmentosum*	334.25	330.10	**325.65**	**324.19**
*Prunus padus*	**401.65**	**403.18**	**402.60**	406.59
*Philadelphus schrenkii*	494.82	**485.27**	496.82	489.04
*Tilia mandshurica*	291.26	**274.06**	291.26	277.87
*Acer triflorum*	95.53	**87.18**	89.18	**87.91**
*Acer mandshuricum*	**99.13**	108.30	101.26	109.24
*Ulmus laciniata*	69.81	74.85	**66.75**	73.39
*Crataegus maimowiczii*	**122.84**	129.91	124.95	128.90
*Malus baccata*	**86.39**	88.95	89.32	91.54

AIC values of the most likely models are shown in bold. Size, Abiotic, Biotic and All factors correspond to models with only tree size, tree size and abiotic factors, tree size and biotic factors, and all factors included, respectively.

**Table 4:** Predictive and discriminative measures (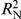 and 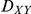) of the generalized linear mixed models of tree survival at the species level.

	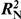	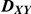
*Corylus mandshurica*	0.037	0.226
*Acer mono*	0.035	0.268
*Acer pseudo-sieboldianum*	0.052	0.319
*Acer barbinerve*	0.060	0.333
*Tilia amurensis*	0.296	0.692
*Pinus koraiensis*	0.044	0.313
*Syringa reticulata*	0.026	0.170
*Ulmus japonica*	0.114	0.444
*Quercus mongolica*	0.080	0.415
*Maackia amurensis*	0.044	0.258
*Fraxinus mandshurica*	0.174	0.539
*Acer tegmentosum*	0.101	0.374
*Prunus padus*	0.085	0.337
*Philadelphus schrenkii*	0.083	0.268
*Tilia mandshurica*	0.304	0.613
*Acer triflorum*	0.279	0.673
*Acer mandshuricum*	0.096	0.440
*Ulmus laciniata*	0.267	0.638
*Crataegus maimowiczii*	0.111	0.383
*Malus baccata*	0.261	0.619

The effects of tree size and abiotic and biotic factors on tree survival varied among these species (Figure S1). Tree size had the most consistent effect on survival across species: for 18 of the 20 species, tree size had the strongest positive effect on survival. The other two species (*Acer tegmentosum* and *Crataegus maimowiczii*) showed no effect of tree size on tree survival. For *C. maimowiczii*, there was also no obvious effect of abiotic and biotic factors on tree survival. In total, eight of the 20 species did not show effects of abiotic and biotic factors on tree survival. Among the other 12 species, three species were affected by only abiotic factors, four species by only biotic factors, and five species by both abiotic and biotic factors. Among the biotic factors that showed effects on tree survival, frequency of neighboring conspecific basal area and total basal area of all neighbors had significant negative effects on tree survival for 4 and 5 species, respectively. One species, *Ulmus laciniata*, showed a significant positive relationship with total basal area of all neighbors.

## Discussion

In this study, we used generalized linear mixed models to conduct a comprehensive analysis of the relative importance of tree size and abiotic and biotic neighborhood variables on tree survival in an old-growth temperate forest in northeastern China. Our results showed that mortality did not occur randomly, since the probability of survival was affected by size, abiotic or biotic variables in nearly all cases. Only one out of twenty species (*C. maimowiczii)* showed no relationships between tree survival and the factors included in the study. In addition, Wang *et al*. [61] analyzed the spatial pattern of mortality within the 5-year period examined, and found that, for most species, dead individuals showed an aggregated spatial pattern at different spatial scales. Together these results indicate that the random mortality hypothesis can be largely rejected for this temperate forest.

### Tree size effects

Although there is no consensus on the shape of the relationship between tree size and survival, empirical and theoretical studies generally suggest that higher mortality rates occur in small tree size classes [9]–[11], [62]. Recently, metabolic ecology theory predicted that tree mortality rates should decrease with tree size, and tree mortality should scale with tree diameter with a constant exponent [62]–[63]. In the CBS forest, tree size did show a strong positive effect on tree survival for most species, but the estimated relationship between tree survival and size (i.e. log(dbh)) varied among species and guilds (Figures 2, 3, and 4 and Figure S1). Moreover, when we classified all individuals into three size classes, we found that effects of tree size on survival turned from strongly positive for small and medium trees, to slightly negative but not significant for large trees (Figure 4), which suggests that the rate at which survival increases with size slows down and levels off, or even declines, at large sizes. This result is consistent with previous studies that found trees did not continue to have higher survival as they became larger [9], [18]–[19]. Therefore, metabolic ecology theory might not be applicable to our temperate forest, as has been previously demonstrated for tropical forests [17]. A possible cause is that metabolic ecology theory assumes that different size classes receive and use the same amount of energy [63]. This assumption may be correct in even-aged forests, but does not extend to natural mixed-aged forests where equal partitioning of energy is unlikely [54].

Size was the strongest predictor of survival at the community level and for nearly all guilds and species analyzed. However, models solely including tree size were often less likely than more complex models with biotic and abiotic predictors. In a study of old-growth conifer forest in the USA, Das *et al.* [5] also found that neighborhood variables, such as conspecific density, improved models used to predict tree mortality. Our results were also consistent with results from a tropical forest: Uriarte *et al*. [9] found that tree survival in a Puerto Rican forest was size-dependent, but also susceptible to crowding effects of neighboring trees, species life-history traits and infrequent disturbances (e.g. hurricanes).

### Abiotic and biotic neighborhood effects

The relative importance of abiotic and biotic factors on tree survival and species coexistence in forest communities has been the subject of a continuous debate, but researchers are now generally convinced that these mechanisms are not mutually exclusive [64]. In the present study, we found that the relative importance of abiotic factors on tree survival tended to be less than that of biotic factors, at least in terms of the variables included in this study. This result may be partially caused by the small spatial variation in abiotic factors in the CBS temperate forest. Topography within the CBS plot is relatively gentle with a maximum difference in elevation of less than 18 m in the 25 ha plot [65]. Therefore, abiotic environmental factors have low spatial heterogeneity, which may explain the relatively small effect of abiotic factors on tree survival. This result is consistent with a recent study of seedling survival in a temperate forest in Japan [66], which found that significant effects of abiotic factors on seedling survival were limited, while effects of biotic variables were more common.

However, the best fit models at the community level and for many of the guilds and species examined tended also to include abiotic factors, indicating that abiotic factors do contribute to patterns of tree mortality. Moreover, our study only included topographical and soil nutrient variables, and may have missed some other important abiotic factors, such as light (although variation in light levels will be captured in part by biotic neighborhood variables). It should also be emphasized that our study is constrained to the plot level (i.e. 25 ha) and that abiotic factors were measured at the 5×5 m scale. At larger and smaller spatial scales, the relative importance of abiotic factors may change.

Among the biotic factors tested, total basal area of neighbors tended to have a negative impact on survival, suggesting competition for resources occurring among trees of all species leads to thinning [67]–[68]. However, the frequency of conspecific neighbor basal area also tended to have a significant negative effect on tree survival, indicating that neighbors of the same species have stronger effects than neighbors of different species, likely due to strong intraspecific competition or natural enemy effects. Although not explicitly addressed in the present study, a number of studies have implicated pathogens as a major driver of negative conspecific effects in plant communities [22], [69]–[70]. Our results are consistent with most previous studies in both tropical [9], [23] and temperate forests [8], [28], [29] that have found negative effects of conspecific neighbors on tree performance. These studies tended to support the idea of negative density- and frequency-dependent effects. As a result, negative density- and frequency-dependence is widely hypothesized to play an important role in population dynamics (e.g. recruitment, growth and survival) of forest trees, and these processes could further contribute to species coexistence in these communities.

### Variation among ecological guilds and species

Considerable differences occurred among ecological guilds in the effects of different factors on tree survival in the CBS temperate forest. For shade-tolerance, we found that survival of light-demanding species and mid-tolerant species were influenced by the biotic, but not the abiotic factors. In contrast, the survival of shade-tolerant species was impacted by both biotic and abiotic factors. This may be because mid-tolerant species and light-demanding species are more sensitive to shading, and thus their patterns of mortality are primarily driven by competition for light (i.e. the biotic neighborhood). In addition, these guilds may be more sensitive to host-specific natural enemies [42], and therefore may be more strongly influenced by their local biotic neighborhoods [71]. Among abundance classes, we found significant effects of abiotic variables only for the common and very common groups. This may reflect that more common species are more strongly impacted by soils and/or topography than less common species; however, because sample sizes were smaller for the less common species groups, our power to detect significant effects of neighborhood variables was reduced relative to analyses of the more common species groups. Previous studies in tropical forests have found that less common species suffer stronger negative effects of conspecific neighbors [23], [43]. However, our study showed that negative effects of conspecific neighbors were strongest for the very common and very rare groups (although not statistically significant for the latter), and weaker, although still significant, for the immediate abundance groups.

Individuals in the three tree size classes also differed strongly in their survival responses. For instance, small trees were more strongly affected by abiotic factors than medium and large trees. Small trees tend to be more susceptible to local environmental conditions than larger trees [44] and can occur in suboptimal sites as a result of seed dispersal patterns. Therefore, small trees in unfavorable habitats may grow more slowly or die off in suboptimal habitats. Thus, larger trees will be found predominantly in their preferred habitat. This is consistent with the idea that habitat filtering at smaller size classes results in adult tree habitat associations [72]. In addition, large trees can change local abiotic properties, such as soil nutrients, over time through decades of feedback via plant litter leaching, litter decomposition and root exudation, as well as associations with micro-organisms [73]–[75].

Total neighbor basal area had negative effects on the survival of small and large trees, but no effects on medium trees. This indicates that the basal area of neighbors influences the survival of even large trees, consistent with results from tropical forests [76]. However, the relative importance of neighbor identity differed between the small and large size classes, with the frequency of conspecifics only having a significant, negative effect on the smaller size class. This suggests that conspecific competition, or apparent competition mediated by natural enemies [77]–[78], was stronger than heterospecific competition for small trees, but became weaker for larger trees. This result is supported by a related study in the CBS temperate plot that found that trees were more regularly spaced in large size classes [53], indicating that large trees have a lower probability of encountering conspecific neighbors than small trees.

We expected species within the same guild to have similar responses to intrinsic and extrinsic factors. However, our results did not meet that expectation. For example, among the six shade-tolerant species in the Aceraceae family, the relative importance of size, abiotic and biotic factors on survival varied dramatically. None of the influential factors had consistent positive or negative effects on tree survival for these six species. *A. tegmentosum* showed no relationship between survival and tree size. Frequency of neighboring conspecifics had negligible effects on survival of *A. pseudo-sieboldianum* and *A. mandshuricum.* Total neighbor basal area only had negative effects on tree survival of *A. mono*. Only half of the six species showed a detectable relationship between survival and abiotic factors. None of these results conformed to the guild-level results for the shade-tolerant guild. The contradiction between the guild-level and species-level analysis may result from the fact that species within a particular guild (e.g. shade tolerant) often varied in terms of other characteristics that influenced survival patterns. For example, the six shade-tolerant species in the Aceraceae family were grouped into three different abundance guilds (two in the very common class, one in the common class and three in the rare class). Also, tree size distributions varied greatly among these species [54]. All these contributed to differences among species. This is consistent with a recent study of seedling survival in a tropical forest where considerable variation occurred among individual species even within the same guild [79].

### Shortcomings of the analysis

Our study presents comprehensive analyses on the relative effects of tree size, abiotic and biotic factors on tree survival in a relatively diverse temperate forest and demonstrates that multiple factors collectively determine a tree's probability of survival. However, the factors included in our study are insufficient to fully understand and predict tree survival in the CBS temperate forest, as suggested by the relatively low predictive and discriminative ability of our models (Tables 2 and 4). Several shortcomings likely affected our results. One is that tree survival is a long-term and complex process that is influenced by many factors, including random events, and our analysis included only a single 5 year census interval. Data are needed on forest dynamics over a larger temporal horizon (e.g. centuries) to capture all the relevant information about survival processes in long-lived trees. Second, other factors that may affect tree survival are not explicitly included in the analysis, such as climate change [19], [80], light [81], pathogen or insect attack [82]–[83], etc. Third, it is difficult to measure the effective neighborhood radius for each tree; hence a fixed maximum distance of influence (i.e. 20 m) was used in the analysis. In addition, by grouping all heterospecific neighbors together, our analysis did not consider species-specific effects on neighbor survival. Previous studies have shown that the effects of different species on plant performance can be asymmetric [9], [25].

### Conclusions

Our study suggests that intrinsic tree size, density- and frequency-dependent effects and niche partitioning with respect to soil and topographic factors contribute to the regulation of the CBS temperate forest community, but the relative importance of these factors varies dramatically among guilds and species. Although the implications of this result for tree species diversity remain to be explored, they imply that attempts to understand, conserve and manage temperate forests should explicitly consider the relative importance of intrinsic and extrinsic factors on forest dynamics. Specifically, if the CBS temperate forest is typical of other temperate forest regions, then one prediction of metabolic ecology theory, that tree mortality should scale with tree diameter with a constant negative exponent [62]–[63], should be rejected. Survival did not continue increasing with tree size and the exponent varied greatly among species. Furthermore, we found strong evidence of negative density dependence, with the frequency of conspecific neighbors having a particularly strong negative impact on survival. This suggests that negative frequency dependence may play a role in the maintenance of diversity in this temperate forest. In addition, our study demonstrates that the relative importance of local-scale variables driving patterns of tree survival varies greatly among species, size classes, guilds and abundance classes in temperate forests. Therefore, predictive models and management decisions should be designed with this variation in mind.

## Supporting Information

Figure S1 **Estimated effects (±2 SE) of abiotic and biotic variables on tree survival for 20 species with >100 individuals in the Changbai temperate forest.** Filled circles indicate significant effects (p<0.05).
(DOC)

Table S1 **Factor loadings of the first two components of the PCA on soil variables from the Changbai temperate forest plot.**
(DOC)

Table S2 **Parameters used in models of tree survival in the Changbaishan temperate forest, northeastern China.**
(DOC)
