# Supplementary material for: Local-Scale Drivers of Tree Survival in a Temperate Forest
Source: PLoS One. 2012 Feb 13;7(2):e29469. doi: 10.1371/journal.pone.0029469 (PMC3278403; doi:10.1371/journal.pone.0029469)
Supplement: Figure S1 — Estimated effects (±2 SE) of abiotic and biotic variables on tree survival for 20 species with >100 individuals in the Changbai temperate forest. Filled circles indicate significant effects (p<0.05). (DOC) [file pone.0029469.s001.doc]

*Corylus mandshurica*

*Acer mono*

*Acer pseudo-sieboldianum*

*Acer barbinerve*

*Tilia amurensis*

*Pinus koraiensis*

*Syringa reticulata*

*Ulmus japonica*

*Quercus mongolica*

*Maackia amurensis*

*Fraxinus mandshurica*

*Acer tegmentosum*

*Prunus padus*

*Philadelphus schrenkii*

*Tilia mandshurica*

*Acer triflorum*

*Acer mandshuricum*

*Ulmus laciniata*

*Crataegus maimowiczii*

*Malus baccata*

**Figure S1.** Estimated **e**ffects (±2 SE) of abiotic and biotic variables on tree survival for 20 species with >100 individuals in the Changbai temperate forest. Filled circles indicate significant effects (p<0.05).
