# Supplementary material for: Local-Scale Drivers of Tree Survival in a Temperate Forest
Source: PLoS One. 2012 Feb 13;7(2):e29469. doi: 10.1371/journal.pone.0029469 (PMC3278403; doi:10.1371/journal.pone.0029469)
Supplement: Table S1 — Factor loadings of the first two components of the PCA on soil variables from the Changbai temperate forest plot. (DOC) [file pone.0029469.s002.doc]

Table S1. Factor loadings of the first two components of the PCA on soil variables from the Changbai temperate forest plot.

| Soil variable | PC1 | PC2 |
| --- | --- | --- |
| pH | -0.339 | -0.154 |
| organic matter | -0.438 | 0.037 |
| available N | -0.389 | 0.278 |
| total N | -0.427 | 0.174 |
| available P | 0.051 | 0.664 |
| total P | -0.411 | 0.232 |
| available K | 0.225 | 0.571 |
| total K | 0.369 | 0.216 |
