# Supplementary material for: Local-Scale Drivers of Tree Survival in a Temperate Forest
Source: PLoS One. 2012 Feb 13;7(2):e29469. doi: 10.1371/journal.pone.0029469 (PMC3278403; doi:10.1371/journal.pone.0029469)
Supplement: Table S2 — Parameters used in models of tree survival in the Changbaishan temperate forest, northeastern China. (DOC) [file pone.0029469.s003.doc]

Table S2. Parameters used in models of tree survival in the Changbaishan temperate forest, northeastern China.

| Parameter | data | | |
| --- | --- | --- | --- |
|  | range | mean | median |
| ***Tree size*** | | | |
| Tree diameter (cm) | 1-158 | 7.62 | 2.1 |
| ***Biotic factors (within 20 m radius)*** | | | |
| Frequency of conspecific basal area | 0-0.67 | 0.07 | 0.02 |
| Total basal area(m^2^) | 2.48-10.6 | 5.49 | 5.43 |
| ***Abiotic factors*** | | | |
| Elevation (m) | 792.1-809.5 | 803.2 | 804 |
| Slope (%) | 0.12-21.69 | 3.16 | 2.48 |
| Aspect | 1.19-359.9 | 215.6 | 266.5 |
| PC1 | -4.92-3.26 | 0 | 0.87 |
| PC2 | -2.87-3.12 | 0 | 0.01 |
